# The Prevalence of Group B Streptococcus Colonization in Iranian Pregnant Women and Its Subsequent Outcome

**Published:** 2013-12-22

**Authors:** Mahboobeh Shirazi, Ezat Abbariki, Ali Hafizi, Fatemeh Shahbazi, Mozhgan Bandari, Ebrahim Dastgerdy

**Affiliations:** 1 Maternal, Fetal and Neonatal Research Center, Tehran University of Medical Sciences, Tehran, Iran; 2 Breast Feeding Research Center, Tehran University of Medical Sciences, Tehran, Iran; 3 Department of Pediatrics, Taleghani Hospital, Shahid Behshti University of Medical Sciences, Tehran, Iran; 4 Department of Biology, Payame Noor University, Iran; 5 Neonatal Intensive Care Unit, Sarem Hospital, Tehran, Iran

**Keywords:** Neonate, Group B Streptococcus, Pregnancy Outcome

## Abstract

**Background:**

Group B streptococcus colonization in pregnant women usually has no symptoms, but it is one of the major factors of newborn infection in developed countries. In Iran, there is a little information about the prevalence of maternal colonization and newborns infected by group B streptococcus. In order to find the necessary information to create a protocol for prevention and treatment of group B streptococcus infection in newborns, we conducted a study of its prevalence among Iranian pregnant women and its vertical transmission to their newborns.

**Materials and Methods:**

This is a cross-sectional descriptive and analytic study performed at Prenatal Care Clinic of the Sarem Hospital from 2009 to 2011. The pregnant women with the gestational age of 35-37 weeks were enrolled in the study. The vaginal culture for group B streptococcus was done for 980 mothers based on our protocol. Among 980 mothers, 48 were shown positive vaginal culture; however, 8 cases among these 48 mothers were positive for both vaginal and urine culture. Babies with mothers showing positive vaginal culture were screened for infection using complete blood count /blood culture (B/C) and C-reactive protein (CRP). Then, a complete sepsis workup was performed for babies with any signs of infection in the first 48 hours after birth, and they received antibiotic therapy if necessary. All collected data were analyzed (SPSS version 15).

**Results:**

Among 980 pregnant women with vaginal culture, 48 cases had positive group B streptococcus cultures among which 8 mothers also had positive group B streptococcus urine culture. Our findings revealed that 22 (50%) symptomatic neonates were born from the mothers with positive vaginal culture for group B streptococcus. About 28 of them (63%) had absolute neutrophil count more than normal, and 4 (9.1 %) newborns were omitted from the study. Therefore, 50% of neonates showed clinical feature, whereas para-clinical test was required to detect the infection for the rest of neonates who showed no signs or symptoms.

**Conclusion:**

The colonization of group B streptococcus in Iranian women is significant, while 50% of newborns from mother with positive vaginal culture were symptomatic after birth; therefore, screening of newborns for group B streptococcus infection is recommended to become a routine practice in all healthcare centers in Iran.

## Introduction

In the recent decade, Group B Streptococcus (GBS) has been one of the common causes of the early onset of sepsis among the newborns, which leads to high rate of morbidity and mortality (1). The incidence of early onset GBS disease is from 1.3 to 3.7 per 10000 live births (2). In addition, GBS is one of the main causes of infection in pregnant women with chorioamnionitis, endometritis, genitourinary tract and surgical wound infection. Genital infection is responsible for almost one-third of preterm deliveries, and GBS produce protease activity resulting to cervical ripening (3).

Most women infected by GBS are asymptomatic, and the organism can be found from their throat, vagina and rectum (4). According to a report by World Health Organization (WHO), the prevalence of GBS colonization in pregnant women is about 5-40% in different countries. Among infected women, 50% showed GBS colonization in their vagina, while the rest revealed infection in their rectum and throat. However, the prevalence of colonization differs based on the age, parity, race, concurrent vaginal yeast colonization, genetic-ethnic factors, socio-economical status, pork consumption and recent sexual intercourse (4, 5).

GBS colonization of the maternal genital tract is related to early onset neonatal sepsis, as a result of vertical transmission before or during labor (6). The rate of vertical transmission of GBS between mothers and their offspring is about 29-85% (mean=51%). This transmission to some extent depends on factors including the severity of maternal colonization in birth canal (4).

The rate of GBS infection in the newborn of colonized mother who has not received antibiotic during delivery is one out of 200, and in cases of receiving antibiotic, it is one out of 4000. In the presence of other predisposing factors like prematurity, maternal fever, premature rupture of membranes (PROM) more than 18 hours, low birth weight and multi parity, the infection rate increases (4). In the USA, the two major prevention strategies for GBS disease include the screening method and the risk-based approach. Pregnant women carrying GBS are offered to take intrapartum antibiotic prophylaxis (7).

The Centers for Diseases Control (CDC) recommended GBS screening for all pregnant women between 35 and 37 weeks of pregnancy, as well as taking intrapartum antibiotic prophylaxis (8, 9). Pregnant women with unknown GBS status should be treated with antibiotic at the time of delivery (4). However, this protocol is not being performed completely in many countries including Iran.

The mortality rate of early onset sepsis has estimated about 50% (9, 10). Furthermore, early onset GBS sepsis leads to a severe neonatal condition, which may result to serious neurological damage. In our country, there is not enough information about maternal colonization and newborn infection with GBS. However, few investigations have been performed. For instance, Fatemi et al. have reported GBS maternal colonization prevalence is about 26.7% among 544 pregnant women in the city of Hamedan, Iran (7). So, we conducted a study of GBS prevalence among Iranian pregnant women and its vertical transmission to their newborns.

## Materials and Methods

This is a cross-sectional descriptive and analytic study performed at Prenatal Care Clinic of the Sarem Hospital in Tehran, Iran in 2011. Vaginal cultures were performed for 980 pregnant women with gestational age of 35-37 weeks. Briefly, two sterile swabs from vagina were taken by a gynecologist and were sent for smear test and culture to the lab. The first swab was used for preparing direct smear and gram staining to detect bacteria, epithelial cells and the number of white blood cells (WBCs). The second swab was cultured for GBS on blood agar, Neisseria on chocolate agar, Gram-negative organism on eosin-methylene blue (EMB) agar and Candida on dextrose agar.

Smear was obtained from β hemolytic colonies on the blood agar. The catalase test was performed on Gram-positive cocci and positive cyclic adenosine mono phosphate (CAMP) colonies.

According to our neonatal intensive care unit (NICU) protocol, complete blood count (CBC), Creactive protein (CRP) and blood count /blood culture (B/C) tests were done for all infants born from mothers with positive history of GBS vaginal colonization (by caesarian section or normal vaginal delivery). If there was any predisposing factor, like premature rupture of membranes (PROM) >18 hours, chorioamnionitis, maternal fever, taking antibiotic during labor, symptomatic newborn, ANC >15000 as a para-clinical infectious predictor, CRP >10 or positive B/C, complete sepsis workup and antibiotics therapy for infants were started.

Newborn with Apgar score <7, meconium aspiration, major anomalies, low birth weight (LBW, <2500 gr), or born from mother with preeclampsia or vaginal bleeding were excluded from our study (4 out of 48). The results were analyzed via SPSS by Chi square, and Fisher’s exact test. Significance level was set at 0.05. The study was approved by The Review Board of Tehran University of Medical Sciences (TUMS) Prenatal Department and all participants gave written informed consent.

## Results

Among 980 pregnant women (aged 19-50 years) with gestational age of 35-37 weeks, 784 (80%) were 25-35 years old. 784 (80%) were prime par and 32 (3.2%) experienced cesarean section. In addition, 48 out of 980 pregnant women had positive vaginal GBS (Table 1), while 8 of these 48 cases showed both positive vaginal and urinary GBS.

**Table 1:** The frequency of vaginal culture in pregnant women


	Number	Percentage (%)

**Candida Spp. **	160	16.3
**Staphylococcus aureus**	15	1.5
**Enterococcus spp.**	21	2.1
**E.coli **	36	3.6
**Klebsiella**	9	0.9
**Non-GBS**	14	1.4
**GBS**	48	4.8
**Total **	303	30.6


Mothers with positive vaginal culture for GBS gave birth to babies who were characterized as 28 neonates (63.6%) with Absolute Neutrophil Count (ANC) more than normal, and 22 neonates (50%) with significant sepsis symptom, including poor feeding, lethargy, hypo-hyperthermia, poor muscle tone, and irritability, while in mothers with negative vaginal culture, only 1% of their babies were symptomatic (p<0.0001, χ^2^=2.27, OR=74.13, CI90: 28.21-194.80, Table 2).

**Table 2:** Correlation between GBS positive vaginal culture and symptomatic neonatal sepsis


	VC [n (%)]		Total [n (%)]
		
	+	-		
		
	+	-	

**SS +**	22 (50)	8 (1)	30 (0.3)
**SS -**	22 (50)	928 (99)	950 (98.7)
**Total**	44	936	980


VC: vaginal culture, SS: sepsis symptom

Also, we found a significant correlation between positive urine culture and positive vaginal culture in our cases. About 17% of mothers with positive vaginal culture had also positive urine culture [p<0.0001, OR=24.3, CI (95): 17.54-32.91].

The gestational age of newborns was between 37 and 39 weeks (38.1 + 1), and their weight ranged between 2500 and 4200 g (3130 + 500).

## Discussion

The overall prevalence for GBS colonization in different countries is reported 5-40% depending on the different regions of the world (4, 8). For example, Grimwood et al. (11) from New Zealand have reported 22%, while Joachim et al. (12) have reported the prevalence of 23% for GBS colonization. Barcaite has shown that the prevalence of GBS colonization in 21 European countries is about 6.5-36% (13). Multiple evidences have shown that the prevalence of GBS colonization is different in each region; for instance, it was reported as 27.6% in Portugal (14), 4.7% in India (15) and 20% in Taiwan (16). In our study, the rate of vaginal colonization was 4.9% which is less than many countries. We only took sample from vagina, but in other studies, the cultures were obtained from vagina, rectum and sometimes throat. In addition, it may relate to other contributory factors, such as the occurrence of colonization in the time interval between culture and delivery, specimen collection, false negative culture due to inadequate swab technique or poor handling, specimen storage conditions, and prolonged transport. Some reports show different positive culture rates in the different culture media (6, 9, 10). Therefore, our results showed that the rate of vaginal colonization in Iran is approximately the same as the other countries or even more. However, more studies are required to determine the specific rate of vaginal colonization.

Although none of mothers in our study had predisposing factor such as PROM, and all of them received antibiotics according to anti-biogram during labor,a newborn in our study had positive B/C. According to the Center for Disease Control (CDC), among 400 newborns in danger of GBS whose mothers has received antibiotic, one newborn showed GBS infection, but in our study, about 50% of the newborns had clinical symptoms which might be due to the severity of maternal colonization and the type of GBS species.

The overall vertical transmission rates of GBS colonization in newborns were reported between 6.4% and 28.4%, while the most studies have indicated colonization rates between 8 and 34.5% (6). A study by Joachim et al. have showed that 10% of infants who were born from GBS positive mothers were infected with GBS (12). Kuhn et al. reported that 0.75 out of 1000 live birth had GBS sepsis (17), which is similar to our study (1 out of 1000). However, another investigation showed that approximately, 1% of the prevalence of sepsis belonged to neonates born from women infected with GBS (6). The causes for differences in prevalence of GBS are identified as follows: density of GBS colonies, intrapartum antibiotic therapy, mode of delivery, and number and time of sampling (6). Most infections in these newborns occur within the first week of life, especially within the first 24 hours, and sepsis was most common symptoms followed by UTI and pneumonia (16, 2).

Early bacterial infections develop neutropenia (18). Neutropenia also was common in our symptomatic neonates due to lack of physiology reserve and immunodeficiency in preterm infants.

## Conclusion

Just like other countries, the maternal colonization with GBS is a common problem in Iran. The rate of GBS infection in Iranian newborns is also like the other countries. In order to obtain more information, we recommend screening for GBS in all pregnant women and a close observation for all their newborns.

It would be efficient to perform screening studies by repeating culture in pregnant women according to microorganism specific enriched media for detecting the GBS species. Early detection results to early treatment by proper antibiotic for newborn infected by GBS. We found that neonates born from women with positive vaginal cultures were more symptomatic than others, so our results suggest that early therapeutic intervention during labor and after birth would be beneficial. However, a sensible long-term plan in order to develop an effective vaccine and its routine usage in healthcare centers would be a real triumph.
